# 

**Published:** 1887-02

**Authors:** 


					﻿fff	2>jcsa ae Sm»d-G<u? Men .’fn£kr.
Vol. n.	FEBRUARY, 1887.	No. 8.
-ff WT*P

.
Subscription, >2.00 a Year, in Advance. — Single Copies, Twenty-fr.e' en-u.
F. E. DANIEL., M D.. Editor and Publisher. AUSTIN. TEXAS;
Wariberc	8«i? Filbert Mrtet P&ltade£g»ha^.
Eugene V<>*: Bog’ ks.’\n.s'; Steam Book and Job PK/NTEK^Aoan*.,
				

